# Texting at the light and other forms of device distraction behind the wheel

**DOI:** 10.1186/s12889-015-2343-8

**Published:** 2015-09-26

**Authors:** James J. Bernstein, Joseph Bernstein

**Affiliations:** Haverford High School, 200 Mill Rd, Havertown, PA 19083 USA; 424 Stemmler Hall, University of Pennsylvania, Philadelphia, PA 19104-6081 USA

**Keywords:** Accident prevention, Adolescent, Cell phones, Distracted driving, Motor vehicle accident, Psychomotor performance, Public policy, Safety, Smartphone, Text messaging, Traffic accidents, Vehicular crashes

## Abstract

**Background:**

Cell phones are a well-known source of distraction for drivers, and owing to the proliferation of text messaging services, web browsers and interactive apps, modern devices provide ever-increasing temptation for drivers to take their eyes off the road. Although it is probably obvious that drivers’ manual engagement of a device while their vehicles are in motion is potentially dangerous, it may not be clear that such engagement when the vehicle is at rest (an activity broadly labeled “texting at the light”) can also impose risks. For one thing, a distracted driver at rest may fail to respond quickly to sudden changes in road conditions, such as an ambulance passing through. In addition, texting at the light may decrease so-called “situational awareness” and lead to driving errors even after the device is put down. To our knowledge, the direct comparison of the rate of device usage by drivers at rest with the rate of device usage by drivers in motion has not been reported.

**Methods:**

We collected information on 2000 passenger vehicles by roadside observation. For the first group of 1000 passenger vehicles stopped at a traffic light, device usage (“texting”, “talking”, “none”), gender of the driver, vehicle type, seatbelt usage and presence of front seat passengers were recorded. For a second set of 1000 vehicles in motion, device usage alone was noted. Statistical significance for differences in rates was assessed with the chi-square test.

**Results:**

We found that 3 % of drivers in motion were texting and 5 % were talking. Among the stopped drivers, 14.5 % were texting and 6.3 % were talking. In the stopped-vehicle set, gender and vehicle type were not associated with significant differences in device usage, but having a front seat passenger and using seatbelts were.

**Conclusions:**

Device usage is markedly higher among drivers temporarily at rest compared with those in motion, and the presence of a front seat passenger, who may help alleviate boredom or reprimand bad behavior, is associated with lower device usage rates among vehicles stopped at a light. These observations may help identify suitable steps to decrease distracted driving and thereby minimize traffic trauma.

## Background

Cell phones are a well-known source of distraction for drivers [1]. According to Strayer et al. [2], the impairments associated with using a cell phone behind the wheel are on par with those of drunk driving, and the US National Safety Council has implicated device usage in 26 % of all vehicular crashes [3]. The proliferation of text messaging services, web browsers and interactive apps make modern devices even more distracting than voice-only cell phones. One may expect that as new features are added in years to come, devices will provide an even greater temptation for drivers to divert their attention from the primary task of operating their vehicles safely [4].

It should be intuitively apparent that manual interaction with a device while driving a moving vehicle will be dangerous. This claim is supported by studies that found that text-messaging was associated with more driving errors [5, 6] and crashes [7]. Yet even with the vehicle at rest, interacting with a device may impose risks: the driver may not be able to respond quickly enough to sudden changes in road conditions, such as an ambulance passing through. In addition, texting may produce a lingering distraction that persists even after the device is put down [8]. This loss of so-called “situational awareness” [9] is reflected in an anecdote shared by a colleague. He reported checking a text message while sitting at the light. After looking up and noticing that the light had turned green, he rushed to accelerate– and promptly rear-ended the car in front of him, which had been slower to take off. Without situational awareness, “the drivers’ eyes may be on the roadway and their hands on the steering wheel, but they may not be attending to the information critical for safe driving”, as Strayer [10] put it.

Interacting with a device with the vehicle temporarily at rest may represent a distinct form of driver distraction. Nonetheless, to our knowledge, a direct comparison of the rate of device usage by drivers at rest with the rate of device usage by drivers in motion has not been reported.

Significant usage differences between drivers at rest and drivers in motion, in turn, might have important implications for possible interventions aimed at decreasing this activity: if nothing else, safety processes that automatically shut down devices when the vehicle begins moving will not address texting at the light.

The research question we therefore address in this study is as follows: What is the incidence of texting with the vehicle at rest as compared with texting while the vehicle is moving? (For brevity, we will designate manual interaction with a device as “texting”, though checking email, web surfing and other related activities would be included in this category.)

To answer the research question, we measured the rate of device usage for a set of vehicles stopped at a busy intersection, and compared it with the rate of device usage in a second set of vehicles that were in motion on the same road at a point just beyond that intersection.

## Methods

Data were collected on both stopped and moving vehicles by a roadside observer stationed on Lancaster Ave and Church Road in Ardmore PA (approximate coordinates: 40.005355,-75.285736) between the hours of 4 pm and 6 pm on weekdays, over a six week period in the summer of 2014. This intersection (https://goo.gl/maps/B0aMw) was selected because it is a bottleneck, with vehicles typically stopped completely for 30 s or more when the light is red. The methods of data collection were modeled after Young et al. [11]. A single observer collected data in batches of 100 vehicles per 1 to 2 h data collection session, to ensure that all vehicles were observed during the afternoon rush hour. There were 1000 vehicles observed while they were stopped at the traffic light. A second group of 1000 vehicles traveling eastward was assessed by an observer standing 25 meters east of the traffic light, yielding a total of 2000 vehicles. The determination of device usage was based on the best judgment of the observer that the driver was manipulating and attending to a palm-sized object. No attempt was made to detect the use of hands-free devices.

### Vehicles at rest

Data were collected from the first six conventional passenger vehicles (sedans, minivans and sport utility vehicles [SUV]) that stopped when the light turned red. For each vehicle, the device usage status of the driver was recorded: “texting” (defined as manipulating a palm-sized object while looking at it), “talking” (defined as holding a device near the head without visual engagement) or “none”. The observer further noted the gender of the driver; the vehicle type (sedan, SUV, or minivan); seatbelt usage status; and whether the vehicle had a front seat passenger or not. Rates were first calculated for overall device usage, and then according to subgroups defined by gender, vehicle type, seatbelt use and front seat passenger status.

### Vehicles in motion

Data were collected by roadside observation of a second set of conventional passenger vehicles in motion at a point approximately 25 meters east of the traffic light. For each vehicle, the device usage status of the driver was recorded. Owing to the speed of the vehicle, the period of observation was necessarily brief, and no attempt was therefore made to record the gender of the driver, vehicle type, seatbelt usage status or the presence of a front seat passenger.

It was assumed that the shorter period of observation for vehicles in motion (1 or 2 s for those in motion vs about 5 s for vehicles at rest) would not meaningfully affect the accuracy of the observations. This assumption is predicated on the belief that device usage is likely to be a prolonged, not fleeting, activity. If so, observations made at a given instant should be comparable to those made over a 5 s period. To ensure the validity of this assumption, a verification study was conducted: 50 consecutive vehicles were assessed by two observers, one stationed at the original point of observation and a second stationed approximately 50 meters further along the road, and the rate of agreement was measured.

Statistical significance for all differences in rates was assessed with the chi-square test at the 0.05 level of significance.

### Ethics statement

This study was reviewed by the Institutional Review Board [IRB] of the University of Pennsylvania and was deemed exempt. As per the IRB protocol, no consent was needed to collect public and anonymous data.

## Results

Among the 1000 stopped drivers, 14.5 % were texting and 6.3 % were talking. The rate of texting and talking among drivers in motion was 3 and 5.5 % respectively (Table 1). The rate of texting differed significantly between groups (*p* < 0.001; chi square statistic = 84.98; two degrees of freedom). In the stopped-vehicle set, gender and vehicle type were not associated with significant differences in device usage, but having a front seat passenger (*p* < 0.001; chi square statistic = 22.52; two degrees of freedom) and using seatbelts (*p* = 0.049; chi square statistic = 6.04; two degrees of freedom) were both associated with lower rates (Tables 2, 3, 4 and 5). In the verification study, the two observers agreed on all 50 observations: 1 driver talking, 49 not using a device.

**Table 1:** Overall device usage

	Stopped	In motion
No device use	792 (79.2 %)	915 (91.5 %)
Texting	145 (14.5 %)	30 (3.0 %)
Talking	63 (6.3 %)	55 (5.5 %)

**Table 2:** Device usage among stopped vehicles, by type

	No device use	Texting	Talking
Sedan (*n* = 700)	79.4 % (556/700)	14.7 % (103/700)	5.9 % (41/700)
SUV (*n* = 259)	78.4 % (203/259)	13.9 % (36/259)	7.7 % (20/259)
Minivan (*n* = 41)	80.5 % (33/41)	14.6 % (6/41)	4.9 % (2/41)

**Table 3:** Device usage among stopped vehicles, with or without a front seat passenger

	No device use	Texting	Talking
No front seat passenger (*n* = 808)	76.2 % (616/808)	16.5 % (133/808)	7.3 % (59/808)
Has front seat passenger (*n* = 192)	91.7 % (176/192)	6.3 % (12/192)	2.0 % (4/192)

**Table 4:** Device usage among stopped vehicles, by driver gender

	No device use	Texting	Talking
Female (*n* = 550)	77.8 % (428/550)	14.7 % (81/550)	7.5 % (41/550)
Male (*n* = 450)	80.9 % (364/450)	14.2 % (64/450)	4.9 % (22/450)

**Table 5:** Device usage among stopped vehicles, by driver seat belt usage

	No device use	Texting	Talking
Seatbelt used (*n* = 936)	80.0 % (749/936)	13.9 % (130/936)	6.1 % (57/936)
No seatbelt (*n* = 64)	67.2 % (43 /64)	23.4 % (15/64)	9.4 % (6/64)

## Discussion and conclusions

The incidence of texting at the light was found to be 14.5 %. This rate is nearly five times that seen among those drivers whose vehicles were moving; it is also more than twice the incidence rate of drivers talking on their device while stopped.

The incidence of device usage among stopped vehicles, we propose, is a potentially more representative metric of driver distraction, as measuring in-motion use alone may understate the problem. Along those lines, drivers who are surveyed and asked if they avoid texting while driving may respond honestly in the affirmative, even if they partake freely in texting while stopped at a light.

Our observation of the high incidence of texting at the light further suggests that many drivers have not stowed their phones while driving. The non-stowed device – near the driver, powered on, and poised to ring or ping– may contribute to the total burden of driver distraction even if the driver does not touch it [12].

The rate of device usage among stopped vehicles contrasted with the rate among moving vehicles offers insights into the psychology of driving. According to the Risk Homeostasis theory, drivers will drive more cautiously when conditions seem to be hazardous and vice versa, to yield an acceptable level of overall risk [13]. The related Task-Difficulty Homeostasis [14] theory maintains that drivers modulate their effort and attention to maintain a given level of challenge; for example, people may drive more slowly on unfamiliar roads. With these hypotheses in mind, the rate of device usage among stopped vehicles may be seen as evidence that some drivers consider texting while stopped to be insufficiently demanding or dangerous.

It should be noted that the Risk Homeostasis theory relates to *perceived* danger. Wilde [15] has asserted that over-estimates of risk can actually lead to improved safety. As an example, he cites the temporary reduction in traffic fatalities after the switch in 1967 to “driving on the right” in Sweden. Yet it also may be the case that an under-estimation of risk has the converse effect. That is, if drivers have miscalculated the risks or demands of texting at the light, they may engage in more texting than they truly “want”, and in so doing expose themselves to levels of danger above the homeostatic set-point.

In the stopped-vehicle set, gender and vehicle type were not associated with significant differences in device usage, but having a front seat passenger and using seatbelts were. The presence of a front seat passenger was associated with a lower rate, suggesting that boredom motivates device usage. This inference is consistent with the theory that a lack of task difficulty liberates drivers to engage in other activities beyond driving itself. [14] Potential disapproval from the passenger may also dissuade device usage. For instance, The National Phone Survey on Distracted Driving Attitudes and Behaviors [16] found that while most respondents felt texting made no difference on their own driving performance, 90 % said they would feel very unsafe as passengers if their driver was using a hand-held device. Disapproval, actual or potential, may curb device use by accompanied drivers.

That device usage was higher among drivers who did not wear a seatbelt may be a manifestation of generalized recklessness in this group. It has been shown that frequent device users also “drive faster, change lanes more frequently, spend more time in the left lane, and engage in more instances of hard braking and high acceleration events” [17]. It would be important to determine the extent to which texting is based on recklessness, as opposed to a mere miscalibration of the task-demands and risks of texting [18]. If the high rates of texting emanate from ignorance about its consequences, education may be an apt remedy. On the other hand, if recklessness were a strong cause, a public awareness campaign aimed at edifying drivers about the dangers of texting may be less effective.

We acknowledge several limitations to this study. First, video recording might have increased the accuracy of the observations. Also, with video we may have been able to capture demographic and other data from vehicles in motion. Video was not employed because of the concern that a noticeable photographer might influence drivers’ actions. We further note that Young et al. [11] reported that live observation was highly consistent between observers, a finding we duplicated in a pilot study.

It also must be acknowledged that the data were collected at a deliberately chosen location, selected because of its typical traffic congestion. Thus the rate of texting at the light seen at this site cannot be generalized, as could be done from a multi-site study [19, 20]. Further, the window of observation for moving vehicles was inherently briefer than for stationary ones. Hence, even though our verification study indicated no differences, we may have undercounted device usage in moving vehicles. We made no attempt to estimate the drivers’ ages, though the finding by Hill et al. [21] that 87 % of college students text at the light suggests that age may be a relevant factor. We further acknowledge that we cannot be certain that drivers who were “manipulating a palm-sized object while looking at it” were necessarily using a device, as opposed to engaging in some less cognitively demanding task. (This distinction may not be critical, as visual distraction has been shown to be the main source of impaired driving performance [22].) Last, the study did not measure the use of hands-free devices, and this omission may introduce an underestimate of the true rate of “talking” in all groups.

In sum, we have found that device usage is higher among vehicles at rest compared with those in motion, and that the presence of a front seat passenger (who may help alleviate boredom or reprimand bad behavior) is associated with lower device usage rates among vehicles stopped at a light. These observations may help identify suitable steps to decrease distracted driving and thereby minimize traffic trauma.

## Abbreviations

SUV: sport utility vehicle
IRB: Institutional Review Board
